# Controlling phase separations and reactions in trapped microfluidic droplets

**DOI:** 10.1038/s41598-024-71586-x

**Published:** 2024-09-09

**Authors:** Sebastian W. Krauss, Matthias Weiss

**Affiliations:** Experimental Physics I, University of Bayreuth, Universitätsstr. 30, 95447 Bayreuth, Germany

**Keywords:** Biophysics, Biological physics

## Abstract

Microfluidics and droplet-based assays are the basis for numerous high-throughput experiments, including bio-inspired microreactors and selection platforms for directed evolution. While elaborate techniques are available for the production of picoliter-sized droplets, there is an increasing demand for subsequent manipulation and control of the droplet interior. Here, we report on a straightforward method to rapidly adjust the size of single to several hundred double-emulsion droplets in a microfluidic sieve by varying the carrier fluid’s salt concentration. We show that the concomitant concentration changes in the droplet interior can drive a reversible demixing transition in a biomimetic binary fluid. As another application, we show that growing and shrinking of trapped droplets can be utilized to achieve a reversible dissociation of double-stranded DNA into single strands, i.e. cycles of reversible DNA hybridization, similar to PCR cycles, can be achieved by reversibly changing the droplet size at constant temperature. Altogether, our approach shows how a simple and temporally tunable manipulation of the size and the chemistry in prefabricated droplets can be achieved by an external control parameter.

## Introduction

The advent of microfluidic devices has fueled a multitude of experiments and applications, both in academia and industry. For example, the possibility to produce isolated and monodisperse picoliter-sized droplets, acting as confinement or microreactor, has facilitated research in soft-matter physics, (bio)chemistry, and biology^1–4^. Prominent applications with connection to the life sciences include, but are not limited to, gene transcription in cell lysates^5,6^, sorting of lysate droplets for directed evolution^7,8^, the self-organization of a mitotic spindle apparatus in confined geometries^9,10^, or applications in personalized medicine^11^. Here, droplets of the desired size are commonly produced by tuning flow velocities in appropriate channel designs, e.g. at T-junctions or via flow focusing^1^, but also droplet formation via microwave heating has been reported^12^. A subsequent agitation or mixing of the droplet interior can be obtained, for example, by incorporating tiny magnetic stir bars^13–16^ during droplet formation and addressing them by an alternating magnetic field.

Changing droplet sizes after the production process, however, is considerably more challenging albeit often desired. For optimizing cell-free gene expression, for example, it turned out to be beneficial to probe different crowding conditions of the lysate by applying osmotic pressure that led to droplet growth or shrinkage^5^. To this end, droplets were moved to sectors in a microfluidic channel at which an adjacent basin with pure water or a saturated salt solution created an osmotic flux via a semipermeable barrier (a thin PDMS wall and an oil film). Upon entering the sector coupled to a saturated salt solution, droplets were seen to shrink by about 25% (from about 27 to 20 μm) within 20 min^5^. However, a quite complex layer composition of the microfluidic device, in combination with a fairly slow shrinking kinetics and a potentially low throughput (droplets need to pass the specific section one by one) limit the versatility of this approach. Furthermore, droplets have been exposed to a static osmotic pressure, e.g. to induce crystallization of colloids in the interior^17,18^. In contrast to the aforementioned approach, however, no temporal changes of the osmotic pressure (e.g. a reversal) was investigated.

Here, we report on an alternative approach that relies on the controlled trapping of up to several hundred double-emulsion droplets. Using water-in-oil-in-water droplets, immobilized in an array of PDMS obstacles within a microfluidic channel, a rapid and reversible change of the osmotic pressure can be achieved, allowing for a cycle of growth and shrinkage of the droplet interior (’osmotic massage’). While previous approaches focused on an irreversible exposure of droplets to a static osmotic pressure to monitor the subsequent relaxation to a new equilibrium, our approach allows for dynamically tuning and reversing the osmotic pressure over time. We show that this approach allows for driving a reversible demixing phase transition of a biomimetic binary fluid inside the droplet. Using an osmotic massage also allowed us to reversibly dissociate DNA double strands into single strands, akin to PCR cycles. These examples highlight the versatility of our approach that may be exploited in many more applications in the future.

## Results and discussion

**Fig. 1:**
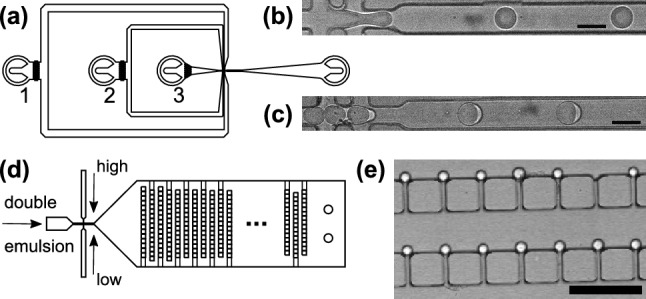
(**a**) Sketch of the microfluidic device used for producing single- and double-emulsion droplets via flow focusing, adapted from^8^. The carrier fluid was injected into inlet 1. Inlets 2 and 3 either supplied aqueous phases or homogeneous PEG-dextran fluids during the formation of single-emulsion droplets or water-in-oil emulsions for double-emulsion production; see also Materials and Methods for details. (**b**, **c**) Representative brightfield images of the production of single-emulsion droplets (water-in-oil) and double-emulsion droplets (water-in-oil-in-water); scale bar 50 μm. (**d**) Sketch of the microfluidic sieve in which double-emulsion droplets are trapped in obstacle fields. Additional inlets for changing the external medium between high and low salt conditions allow for altering the ambient osmotic pressure on trapped droplets, hence changing the intra-droplet milieu. (**e**) Representative brightfield image of double-emulsion droplets, trapped at a row of obstacles; scale bar 200 μm.

**Fig. 2:**
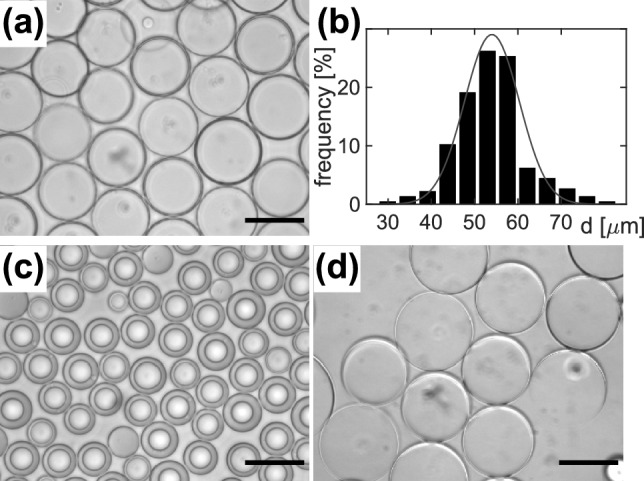
(**a**) Representative brightfield image of double-emulsion droplets in 150 mM NaCl solution; scale bar 50 μm. (**b**) Under these conditions, droplets are fairly monodisperse (black bars), featuring roughly a Gaussian distribution with a mean diameter $d\approx 55$ μm (red line). (**c**) When exposing double-emulsion droplets to 1 M NaCl, the mean diameter of the inner aqueous phase decreased to $d\approx 17$ μm. (**d**) In pure water, droplets increased to a mean diameter of $d\approx 58$ μm; scale bars $50$ μm.

After producing double-emulsion droplets (see Fig. 1 and Materials and Methods for details), we first examined their size distribution as well as their response when exposing them to an osmotic pressure in a simple bulk assay. At 150 mM NaCl, double-emulsion droplets featured a fairly homogenous size with a mean diameter of 55 μm for the inner core (see Fig. 2a, b). Shifting the droplets to 1 M NaCl, applying a strong osmotic imbalance, decreased the inner aqueous droplet considerably (Fig. 2c), leading to a strongly reduced mean diameter of about 17 μm at steady state. In contrast, immersing droplets in pure water led to a swelling (Fig. 2d) with a slightly larger mean diameter of the inner droplet (58 μm) at steady state. Both observations confirm that the oil phase that separates the interior aqueous phase from the surrounding medium acts similar to a semipermeable barrier, i.e. it is permeable for small water molecules, hence relaxing the osmotic pressure, whereas larger and charged molecules are trapped in the inner aqueous phase as their partition coefficient to the oil phase is markedly lower^19^.

**Fig. 3:**
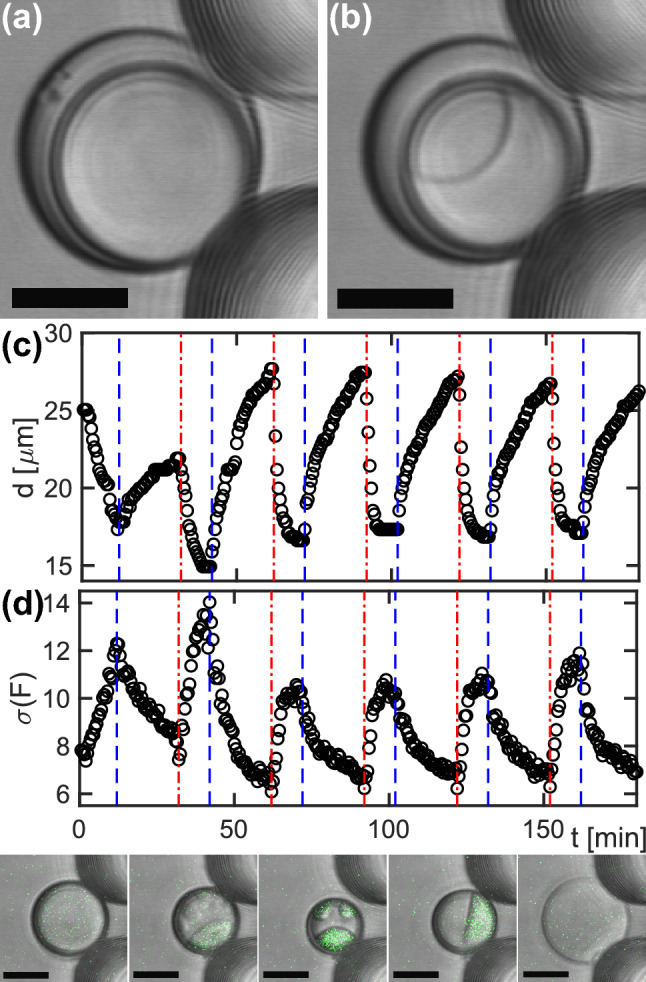
(**a**, **b**) Representative brightfield images of a single double-emulsion droplet, hosting a binary PEG-dextran fluid as inner aqueous core, trapped in the microfluidic sieve. At 150 mM NaCl in the external medium, the PEG-dextran fluid is homogeneous (subfigure **a**). After shifting to 1 M NaCl, the same droplet has crossed the binodal and shows an unmixing into two adjacent phases (subfigure **b**); scale bar $15$ μm. (**c**) Corresponding time evolution of the diameter *d* of a single droplet. Periodically flushing the channel with pure water or 1 M NaCl solution (indicated by blue dashed and red dash-dotted lines) yielded a rapid and periodic cycling of the inner droplet’s diameter (black symbols) after an initial equilibration phase ($t<30$ min). While the minima of *d* are in agreement with observations from a bulk assay at 1 M NaCl (cf. Fig. 2c), the maxima remain below the anticipated value of $58$ μm (cf. Fig. 2d) due to an incomplete relaxation before initiating the next medium change. (**d**) The standard deviation of pixelwise fluorescence values, $\sigma (F)$, determined from FITC-coupled dextran inside the droplet, yield a simple measure for the reversible emergence of a phase separation of PEG- and dextran-enriched phases upon crossing the binodal: Droplets with a large diameter feature a homogeneous fluid with similar fluorescence values in every pixel, whereas droplets with low diameters host a phase-separated fluid with distinct pools of bright and dark pixels, leading to elevated values of $\sigma (F)$. The lower panel shows representative fluorescence images (superimposed in green to brightfield snapshots) after switching to 1 M NaCl (images 1–3) and reverting to pure water (images 4, 5); see also supplementary movie 1. Note that when crossing the binodal, several dextran-rich phases may initially appear (cf. images 2, 3), which quickly merge into a single phase; scale bars 20 µm.

Based on these observations, we reasoned that not just the droplet volume but due to a change in the amount of water also the concentrations of solutes inside the droplet can be tuned, hence allowing us to drive a rapid and reversible phase separation of the Flory-Huggins type in the inner aqueous phase. To probe this, we used a well-characterized biomimetic binary fluid^20^, composed of poly-ethyleneglycol (PEG) and dextran in MilliQ water (see Materials and Methods for details). Being a homogeneous fluid above the binodal, PEG- and dextran-rich phases segregate upon crossing the binodal^20^, leading to phase-specific refractive indices that can be detected optically. Plotting PEG concentration vs. dextran concentration, the binodal has a roughly hyperbolic appearance^20^, i.e. extracting water from the droplet leads to an increase of both concentrations. As a result, the binodal is crossed along the diagonal and segregated PEG-rich and dextran-rich phases are observed. We therefore produced double-emulsion droplets with the inner aqueous phase being a binary PEG-dextran fluid and trapped these droplets in a microfluidic sieve (cf. Fig. 1d, e). Using pure water or a 150 mM NaCl solution as external medium, the droplets’ inner phase was in the mixed state (Fig. 3a), whereas switching to 1 M NaCl decreased the droplet size so that the binary fluid crossed the binodal toward the unmixed state. As a result, the droplet displayed two adjacent phases in which either PEG or dextran were in the majority (Fig. 3b), with the phase boundary being clearly visible.

Switching the external medium periodically, i.e. flushing the channel periodically with pure water and 1 M NaCl solution, the inner droplet diameter was seen to reversibly grow and shrink after an initial equilibration during the first period (Fig. 3c). Size adaption upon flushing the microfluidic sieve with 1 M NaCl was very rapid with a typical shrinkage to about half of the diameter within 5 min or less. Compared to earlier approaches^5^, that allowed for a shrinking of droplet diameters by 25% within 20 min, this shrinkage kinetics is very rapid. Droplet growth after shifting the external medium back to water took somewhat longer, due to a lower concentration difference and hence a lower osmotic pressure. Moreover, swollen droplets did not reach the anticipated steady-state diameter of 55 μm since switching of the external medium to 1 M NaCl was triggered well before complete equilibration.

Since the droplet volume is coupled to its diameter ($V\sim d^3$), a diameter increase from $d=17$ μm (at 1 M NaCl) to $d=28$ μm (at 150 mM NaCl) within one cycle is associated with a roughly 4.5-fold change in volume. Given that droplet growth and shrinkage is driven by water uptake and loss, respectively, the concentration of PEG/dextran can therefore be expected to also change about 4.5-fold during one cycle. For an estimate in absolute numbers, one may start from the initial concentration of PEG and dextran in pure water (each 9% wt/wt, see Materials and Methods), used for producing the inner droplet. These aqueous droplets maintained their diameter during the encapsulation process (performed at 150 mM NaCl, see Materials and Methods), leading to a mean droplet diameter of $d=55$ μm (Fig. 2b). A comparison to $d=17$ μm in the presence of 1 M NaCl therefore indicates a roughly 34-fold volume reduction or concentration increase (from about 0.1 g/ml to 3.4 g/ml for each, PEG and dextran).

To monitor the reversible demixing transition, we added minor amounts of FITC-labeled dextran to the inner droplet and extracted the resulting pixelwise fluorescence. In particular, we estimated the variation of recorded fluorescence values in the inner droplet by calculating the standard deviation $\sigma (F)$ over all pixels in a single frame as a simple readout (see Fig. 3d): In the mixed state, the dye was homogeneously dispersed over the inner droplet, yielding a histogram of pixelwise fluorescence values that featured a single peak and a moderate width (i.e. standard deviation). Upon droplet shrinkage the binary fluid crossed the binodal, resulting in the emergence of one or few fluorescent blobs in the dextran-rich phase that eventually converged to a single phase. Therefore, a bimodal distribution of fluorescent pixels with high and low values in the dextran- and PEG-rich phase emerged, yielding increased values for $\sigma (F)$. In line with the periodically varying droplet diameter (Fig. 3c), we observed a periodic increase and decrease of $\sigma (F)$ when cycling the osmotic pressure (Fig. 3d), reflecting the reversible emergence of the demixing phase transition. The associated time course of the reversible demixing transition when applying an osmotic massage to the droplet can also be viewed in the supplementary movie 1. Therefore, our approach can not just be used to tune the size of droplets but also to induce or revert an unmixing phase transition in the droplet interior. As for a practical application, we would like to highlight that it has already been demonstrated that this very phase separation can be used to facilitate enzymatic reactions^21^, suggesting that also other reaction schemes might benefit from such a phase environment. In particular, we envisage that controling phase separations can be a versatile tool to control the dynamics of reactions in the droplet interior with spatiotemporal precision. Moreover, our approach may be a versatile tool for the active field of phase separations in biology, e.g. for studying the formation of condensates of cytoplasmic proteins^22,23^ or the formation membraneless organelles^24^.

**Fig. 4:**
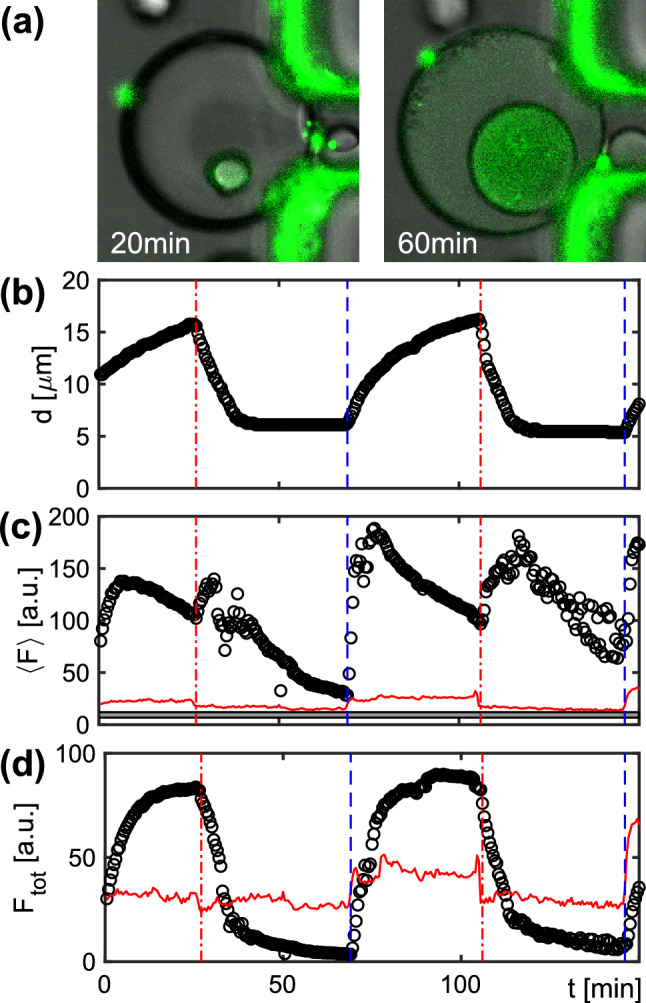
(**a**) Representative brightfield images of a double-emulsion droplet, hosting an alkaline aqueous solution supplemented with a DNA ladder and SYBR Green I (fluorescence superimposed in green). When immersed in a 1 M NaCl solution, the small droplet has a pH$\approx$14 at which DNA is preferentially present as single strands that form only few fluorescent complexes with SYBR Green I (left). Upon swelling the droplet in pure water (right), the pH drops and the DNA re-anneals to double strands, and forms plenty of fluorescent complexes with SYBR Green I. Please note that the oil phase around the inner droplet also has a measurable fluorescence. The highly fluorescent structure next to the droplet is due to SYBR Green I that partitioned from the surrounding into the adjacent PDMS pillar of the microfluidic sieve, a process that was seen to occur independent of the actual osmotic pressure. (**b**) Periodically flushing the channel with pure water or 1 M NaCl solution (indicated by blue dashed and red dash-dotted lines), the droplet diameter is seen to shrink and grow. (**c**) The average fluorescence per pixel in the inner droplet, $\langle F\rangle$, here displayed as 8-bit grey values, follows the volume changes induced by the osmotic massage (black symbols). Imaging during the shrinkage process occasionally suffered from droplets migrating slightly out of focus. While the total fluorescence was hardly affected by this (cf. subfigure **d**), the average fluorescence per pixel was more sensitive, yielding visible variations in $\langle F\rangle$. The corresponding values for pixels in the oil phase show a significantly lower value due to a lack of double-stranded DNA in this phase. (**d**) The total fluorescence of the inner droplet, $F_{\text {tot}}$, here shown as the product of droplet area and mean pixel intensity (in arbitrary units), is a proxy for the total amount of double-stranded DNA. As expected, $F_{\text {tot}}$ shows a strong increase upon swelling the droplet, reflecting the increase in double-stranded DNA. Upon decreasing the droplet, also $F_{\text {tot}}$ is reduced to the initial levels, i.e. the DNA is reversibly switched between the single- and double-stranded form. In comparison, $F_{\text {tot}}$ in the oil phase is markedly lower and changes less during the osmotic massage.

As another application of an osmotic massage of droplets, we considered the melting and re-annealing of double-stranded DNA. In most applications, e.g. in the polymerase chain reaction (PCR), a reversible melting of double-stranded DNA into single strands is accomplished by changing the temperature. However, hydrogen bonds between complementary nucleotides in helical DNA structures can also be broken by chemical means, e.g. by increasing the hydroxide concentration at high pH. We therefore reasoned that swelling and shrinking of droplets can be used to vary the pH, hence resulting in a reversible melting and annealing of double-stranded DNA. A convenient reporter for this transition is the nucleic acid stain SYBR Green I, which is widely used for quantitative PCR, that becomes fluorescent upon binding to double-stranded DNA^25^. Following this rationale, we used a simple DNA ladder in 8.5 M NaOH (pH $\approx$ 14) for the inner aqueous droplet. We then monitored the droplet diameter and the pixelwise fluorescence of SYBR Green I when switching the surrounding from a 1 M NaCl solution to pure water and back (see Fig. 4a for representative snapshots). When being immersed in pure water, the inner droplet is expected to swell, leading to a lower pH. While swelling, the pH in the inner droplet decreases and DNA strands start to re-anneal, i.e. more complexes with SYBR Green I are formed. Therefore, an increase of the total fluorescence of the droplet is expected.

In line with our expectations, we observed the diameter of the inner aqueous droplet to grow and shrink after the respective medium exchange (Fig. 4b). The average fluorescence per pixel in the inner droplet also showed marked variations during droplet swelling and shrinking: After shifting to pure water, a sudden increase of the average pixel fluorescence is seen, followed by a slow decrease (Fig. 4c). This decrease is not unexpected as the number of fluorescent complexes gets successively diluted as the droplet grows. The total fluorescence of the droplet, however, should report on the net increase of fluorescent SYBR Green complexes. In line with this notion, the product of the average pixel fluorescence and the droplet area in the image (basically a smoothened sum of all pixel intensities) shows rapid relaxations to high and low plateau values (Fig. 4d), hence confirming that more double-stranded DNA is seen when droplets have grown. For comparison, we would like to mention that the residual fluorescence in the oil phase was considerably lower in both quantities; fluorescence in the outer aqueous phase was virtually zero. Repeating the experiment with SYBR Green in the absence of DNA, resulted in a total fluorescence that was comparable to that of the oil phase. We therefore conclude that the observed effect is specific and reports on the reversible melting of double-stranded DNA. Based on this proof-of-principle experiment, numerous future applications can be envisaged. For example, a higher yield of correctly folded DNA origamis may be achieved via cycles of association and dissociation that reduce the amount of kinetically trapped states^26,27^. Moreover, the spatial confinement in droplets in conjunction with an osmotic massage may be used for the formation of micron-sized DNA-based hydrogels^28^.

In conclusion, we have introduced here a straightforward and versatile approach for altering the size of picoliter-sized droplets in a microfluidic sieve with a rapid response. The approach also allows for driving reversible phase transitions in binary fluids, i.e. a reversible demixing transition upon crossing the binodal. As a proof-of-principle application, we have also demonstrated that a reversible melting of DNA by varying the droplet’s internal pH is possible. We expect the method to be versatile in the wider context of microfluidic applications, supposedly with a predominant usage in the realm of biological and biomimetic systems.

## Materials and methods

Microchannels were produced by standard soft lithography as described before^29^. In particular, the master mold was manufactured using a negative photoresist (SU-8 2015; microchem) spread by spin coating on a silicon wafer to a height of 20 μm and 100 μm for droplet production and capture, respectively. After soft-baking on two hotplates ($65$ °C for 2 min and $95$ °C for 5 min) the channel design was directly written with a microwriter (MicroWriter ML3 Baby$+$; Durham Magneto Optics). After 3 min in developer bath and a post exposure bake ($65$ °C for 1 min and $95$ °C for 5 min), the unexposed photoresist was removed by successively washing the wafer with developer (mr-Dev 600) and isopropanol, followed by drying with compressed air. Polydimethylsiloxane (PDMS) based microfluidic chips were then drawn from the master. To this end, PDMS (Sylgard$^{\text {TM}}$ elastomer 184; DOWSIL) was mixed with curing agent in a 10:1 ratio and poured onto the wafer after 2 min of mixing. Degassing was performed two times for 2 min in a Duran desiccator (Schott). After baking in the oven at $75$ °C for 3.5 h, the cured elastomer was removed from the wafer with a scalpel. Inlets and outlets were punched into the PDMS with a stainless steel puncher (Harris Uni-Core, 1.2 mm). The device was bonded to a microscope glass slide (cleaned by sonication in isopropanol for 10 min and dried in nitrogen flow) following a plasma exposure for 20 s at 122 Pa. Bonding was strengthened by storage at $75$ °C in the oven for 45 min.

For achieving the desired surface properties of PDMS channels, we adapted previous protocols^8^: Hydrophobic surfaces were obtained by filling channels with 1% trichloro(1H, 1H, 2H, 2H-perfluorooctyl)silane in the fluorinated oil HFE 7500 directly after plasma treatment and bonding of the PDMS to glass for 20 min at $70$ °C. The filled chip was then heated to $75$ °C for 20 min on a hotplate to evaporate the supernatant, covering the PDMS walls stably with fluorinated hydrocarbons. For hydrophilic surfaces, a solution of poly(diallyldimethylammonium chloride) (200–359 kDa, 2 mg/ml in 0.5 M NaCl) was injected into channels, creating a stable layer of positive charges on the PDMS surface. This step has to be done directly after the bonding without any further heating in between. After 10 min the solution was flushed out with 0.1 M NaCl, followed by an injection of a solution of poly(styrene sulfonate) (70 kDa, 2 mg/ml in 0.5 M NaCl) to create stable negative charges on the positive polycations. After 10 min this solution was flushed out with MilliQ water and all inlets were sealed with tape to prevent evaporation.

Double-emulsion droplets were produced in two consecutive steps, both using the same flow-focusing device (Fig. 1a), according to the approach described in Ref. ^8^: First, water-in-oil droplets were produced in fluorinated oil HFE 7500 (3M) with 1% (w/w) of the fluorosurfactant RainDance EA (surfactant 008; RAN biotechnologies) by flow focusing (see Fig. 1b for a representative experiment). Using high-precision syringe pumps (CETONI NEMESYS low pressure module V2), connected to the device by PTFE teflon tubings, the aqueous phase was injected into the hydrophobically rendered chip at a flowrate of 40 μl/h; the oil phase had a flowrate of 500 μl/h. The obtained single-emulsion droplets were then introduced into a hydrophilic channel (flowrate 70 μl/h), with two successive flow focusing steps. First, droplets were separated using fluorinated oil HFE 7500 (3M) with 1% (w/w) of the fluorosurfactant RainDance EA (flowrate 70 μl/h) followed by the encapsulation with 150 mM NaCl with 1% (w/w) of the surfactant Tween 80 (flowrate 500 μl/h; see Fig. 1c for a representative experiment).

Double-emulsion droplets, immersed in 150 mM NaCl, had a typical diameter in the range of 50 μm (cf. Fig. 2). Injecting them into a hydrophilically rendered microfluidic device with a sieve-like design (Fig. 1d) and keeping them exposed to a constant flow (20 μl/h), led to a trapping of droplets (see Fig. 1e for an example). Specifically, a 2 mm wide and 100 μm high channel hosted 24 rows of square pillars (edge length 100 μm), separated by 100 μm wide gaps. Within each row, the distance between pillars was successively reduced from 10 μm down to 1 μm after every fourth row, resulting in a successively finer mesh for immobilizing droplets. Double-emulsion droplets were too big to pass the gaps between pillars, and hence were pressed and immobilized at the gaps between pillars. Additional influx channels allowed a quick switching between high and low salt concentrations of the external medium to alter the osmotic pressure on the interior aqueous phase of the double-emulsion droplets. Droplets were seen to be stable in a wide range of salt concentrations (0-3 M NaCl).

For studying the demixing transition of a biomimetic binary fluid, the inner droplet of double-emulsion droplets consisted of MilliQ water with 9% (w/w) poly(ethyleneglycol) (PEG 10 kDa; Sigma-Aldrich), 9% (w/w) dextran (6 kDa; Sigma-Aldrich), and 0.1% (w/w) fluorescein-5-isothiocyanate (FITC) labeled dextran (10 kDa; Sigma-Aldrich). Since this mixture is near to the binodal but still in the mixed regime, it was possible to directly use the binary fluid for producing the inner aqueous phase of double-emulsion droplets as described above. Double emulsions were imaged in brightfield and fluorescence mode with a Leica SP5 II confocal laser scanning microscope using a HCX PL FLUOTAR L 20x/0.40 dry objective. FITC-labelled dextran was excited at 496 nm with an Ar laser and the resulting fluorescence was collected in the range 510–550 nm. The osmotic massage was monitored for 2–3 h at room temperature (about $20$ °C) with two frames per minute. Here, trapped droplets were periodically exposed to 1 M NaCl solution (10 min) and pure water (20 min) at a flow of 20 μl/h.

For hybridization experiments, the inner aqueous phase of double-emulsion droplets consisted of 2.5 ng/ml of a DNA ladder with 10–300 base pairs (Gene Ruler Ultra low range 10–300 bp; ThermoFisher Scientific) and 1 volume% of SYBR Green I Nucleic Acid Gel Stain (Hycultec HY-K1004) in 8.5 M NaOH. Imaging was done as described above with the exception that the fluorescence of SYBR green was collected in the range 510–600 nm. The flow protocol consists of periodically changing between 3 M NaCl solution (40 min) and MilliQ water (40 min), both being supplemented with 1vol% of SYBR green.

## Supplementary Information

Supplementary Information 1.

Supplementary Information 2.

## Data Availability

Data sets generated during the current study are available from the corresponding author on reasonable request.
